# *Atp6v1h* Deficiency Blocks Bone Loss in Simulated Microgravity Mice through the *Fos-Jun-Src-Integrin* Pathway

**DOI:** 10.3390/ijms25010637

**Published:** 2024-01-04

**Authors:** Zanyan Zhao, Xiangpu Wang, Yu Ma, Xiaohong Duan

**Affiliations:** State Key Laboratory of Oral & Maxillofacial Reconstruction and Regeneration, National Clinical Research Center for Oral Diseases, Shaanxi Key Laboratory of Stomatology, Department of Oral Biology, Clinic of Oral Rare and Genetic Diseases, School of Stomatology, The Fourth Military Medical University, Xi’an 710032, China; zyz135921@163.com (Z.Z.); wxp901120@163.com (X.W.); 18391689867@163.com (Y.M.)

**Keywords:** osteoporosis, *Atp6v1h*, *Fos-Jun-Src-Integrin* pathway, tail suspension model

## Abstract

The microgravity conditions in outer space are widely acknowledged to induce significant bone loss. Recent studies have implicated the close relationship between *Atp6v1h* gene and bone loss. Despite this, the role of *Atp6v1h* in bone remodeling and its molecular mechanisms in microgravity have not been fully elucidated. To address this, we used a mouse tail suspension model to simulate microgravity. We categorized both wild-type and *Atp6v1h* knockout (*Atp6v1h*^+/-^) mice into two groups: regular feeding and tail-suspension feeding, ensuring uniform feeding conditions across all cohorts. Analysis via micro-CT scanning, hematoxylin-eosin staining, and tartrate-resistant acid phosphatase assays indicated that wild-type mice underwent bone loss under simulated microgravity. *Atp6v1h*^+/-^ mice exhibited bone loss due to *Atp6v1h* deficiency but did not present aggravated bone loss under the same simulated microgravity. Transcriptomic sequencing revealed the upregulation of genes, such as *Fos*, *Src*, *Jun*, and various integrin subunits in the context of simulated microgravity and *Atp6v1h* knockout. Real-time quantitative polymerase chain reaction (RT-qPCR) further validated the modulation of downstream osteoclast-related genes in response to interactions with *ATP6V1H* overexpression cell lines. Co-immunoprecipitation indicated potential interactions between ATP6V1H and integrin beta 1, beta 3, beta 5, alpha 2b, and alpha 5. Our results indicate that *Atp6v1h* level influences bone loss in simulated microgravity by modulating the *Fos*-*Jun*-*Src*-*Integrin* pathway, which, in turn, affects osteoclast activity and bone resorption, with implications for osteoporosis. Therefore, modulating *Atp6v1h* expression could mitigate bone loss in microgravity conditions. This study elucidates the molecular mechanism of *Atp6v1h*’s role in osteoporosis and positions it as a potential therapeutic target against environmental bone loss. These findings open new possibilities for the treatment of multifactorial osteoporosis.

## 1. Introduction

Osteoporosis represents a critical and escalating public health concern, marked by systemic degradation of bone mass and microarchitecture, culminating in an enhanced risk of fragility fractures [1]. Given the demographic shift towards an aging population in numerous countries, osteoporosis is poised to emerge as a formidable global challenge.

Contemporary research suggests that osteoporosis etiology is multifaceted, encompassing a complex interplay of environmental, endocrine, and genetic elements. Environmental factors contributing to osteoporosis include: (a) Nutritional issues, such as deficiency in vitamin D, inadequate sunlight exposure, and unbalanced diets [2]. (b) Sedentary lifestyles, alterations in gravitational forces, and excessive mechanical stress, with a notable example being the bone loss observed in microgravity environments. This was first recorded in the mid-1970s by Skylab astronauts, who experienced a monthly bone mass reduction of 1–2% compared to pre-flight and ground-based controls [3,4]. Subsequent investigations have highlighted the activation of the RANKL/RANK/OPG, PPARγ, and PKA/CREB signaling pathways in microgravity, whereas the Wnt/β-catenin and BMP/Smad pathways are suppressed, leading to enhanced bone resorption and inhibited bone formation [5,6,7,8,9]. (c) Prolonged use of medications like anticonvulsants, glucocorticoids, sedatives, or chemotherapeutic agents [10]. (d) Lifestyle factors such as alcohol consumption, caffeine intake, and smoking [11]. Endocrine factors predominantly involve alterations in estrogen levels due to aging, menopause, and psychological stress [12,13].

Genetic factors play a pivotal role in modulating bone mineral density (BMD), significantly influencing the progression of osteoporosis by affecting bone size and quality. Investigations involving twins and familial studies have demonstrated that genetic factors account for approximately 50% to 85% of osteoporosis etiology [14,15]. In clinical settings, bone density is a critical diagnostic and therapeutic marker for osteoporosis [16]. Therefore, a comprehensive understanding of the molecular mechanisms by which genetic and environmental factors influence bone density is imperative for the development of efficacious treatments for osteoporosis in the future.

*ATP6V1H*, encoding the H subunit of V-type proton ATPase, plays a pivotal role in bone homeostasis [17]. Our team was the first to identify *ATP6V1H* as a causative gene for osteoporosis in 2016. We employed CRISPR/Cas9 technology to specifically delete the *Atp6v1h* gene and found that *Atp6v1h*^+/-^ mice have pronounced bone matrix loss and compromised bone formation by regulating the balance between osteoclast and osteoblast through the TGF-β1 pathway [18], or the proliferation and differentiation of bone marrow stromal cells through interactions with TGF-β receptor I and the AP-2 complex [19]. In zebrafish, loss-of-function mutations in atp6v1h lead to a marked decrease in the population of mature calcified bone cells [20].

Traditionally, osteoporosis research has predominantly focused on the individual impacts of either environmental or genetic factors on bone mass. However, there is a paucity of studies investigating the synergistic effects of these factors on the onset and progression of osteoporosis. The mouse tail suspension method, an internationally acknowledged technique for simulating microgravity, offers the advantages of simplicity and scalability [21]. In our study, we utilized *Atp6v1h* deficiency mice and applied the mouse tail suspension method to simulate microgravity conditions. We compared the bone characteristics of both wild-type (WT) and *Atp6v1h*^+/-^ mice under these simulated conditions. This approach potentially lays new theoretical groundwork for elucidating the pathogenesis of osteoporosis, considering both environmental and genetic factors, and it may pave the way for novel osteoporosis drug development.

## 2. Results

### 2.1. The Mouse Tail Suspension Model Was Successfully Established, and the Bone Mineral Density of Mice Was Decreased

**Figure 1:**
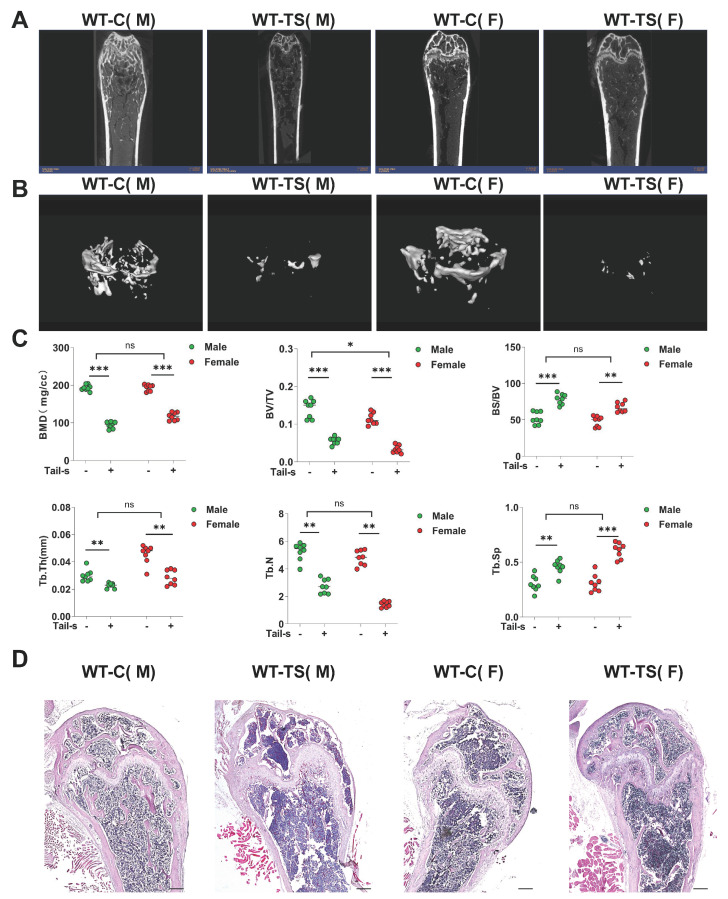
Mouse tail suspension model can reduce bone mineral density in wild-type mice. (**A**) The two-dimensional micro-CT scan images of the femur from different groups. (**B**) Three-dimensional images of micro-CT scan of the femur. (**C**) Statistics of micro-CT-related parameters: BMD (bone mineral density), BV/TV (bone volume ratio), BS/BV (bone surface area to bone volume ratio), Tb. Th (trabecular thickness), Tb. N (trabecular number), Tb. Sp (trabecular bone separation) (All data represent mean ± SD, *** *p* < 0.001, ** p < 0.01, * *p* < 0.05, ns: no significant difference). (**D**) H&E staining of mouse femur tissue (scale bar: 200 μM). Each experimental group consisted of 8 mice; all mice were 3 months old.

Micro-computed tomography (Micro-CT) analysis was performed on femurs from both male and female WT mice across control and suspension cohorts. The bidimensional Micro-CT representations of these femurs elucidated a diminution in bone trabecular structure and a concomitant attenuation of cortical bone thickness in the suspension group, relative to controls (Figure 1A). Additionally, tridimensional Micro-CT imaging further delineated a notable reduction in trabecular number, a decrease in trabecular thickness, and an augmentation in trabecular separation within the suspension cohort as compared to the control group (Figure 1B).

A comprehensive statistical analysis of bone-related parameters was conducted (Figure 1C). The Student’s t-test facilitated the evaluation of differential bone parameters between the regular and suspension cohorts. Additionally, a two-way ANOVA was employed to discern sex-specific variations in bone alterations between male and female mice. Notably, in the suspension group of both genders, there was a significant reduction in bone mineral density (BMD), bone volume fraction (BV/TV), trabecular thickness (Tb. Th), and trabecular number (Tb. N) compared to the control group (*p* < 0.05). Conversely, parameters such as bone surface to volume ratio (BS/BV) and trabecular separation (Tb. SP) exhibited significant increments (*p* < 0.05), indicative of a pronounced osteoporosis-like condition. Post-suspension, no substantial gender-based disparities in bone parameters were observed, except in the case of Tb. Th (*p* > 0.05). Consequently, the tail suspension model implemented herein effectively induced significant bone loss in mice.

Hematoxylin and Eosin (H&E) staining was meticulously performed on femurs from both male and female WT mice across regular and suspension groups, ensuring the structural integrity of all specimens for analysis (Figure 1D). Relative to the regular group, male mice in the suspension cohort manifested several critical changes: the cortical bone and cartilage tissues were markedly thinner, coupled with a pronounced decrease in the number of bone trabeculae. Furthermore, both trabecular thickness and separation were notably augmented.

Comparative analyses between female suspension and non-suspension WT mice revealed alterations akin to those observed in male counterparts: a marginal thinning of cortical and cartilage tissues, coupled with a discernible decrease in the relative volume, number, and thickness of bone trabeculae, and an increased presence of adipocyte-like vacuoles in the bone marrow. Further morphological comparison of femurs between male and female mice elucidated that the femoral heads in females typically exhibited a smaller size, with relatively thicker cortical bone and cartilage tissues. Given the consistent trend in femoral alterations post-suspension across genders in WT mice, subsequent experiments were predominantly focused on male mice as representative subjects.

### 2.2. The Decrease in Bone Mineral Density in WT Mice in the Simulated Microgravity Environment Was Due to the Increased Number and Activity of Osteoclasts

**Figure 2:**
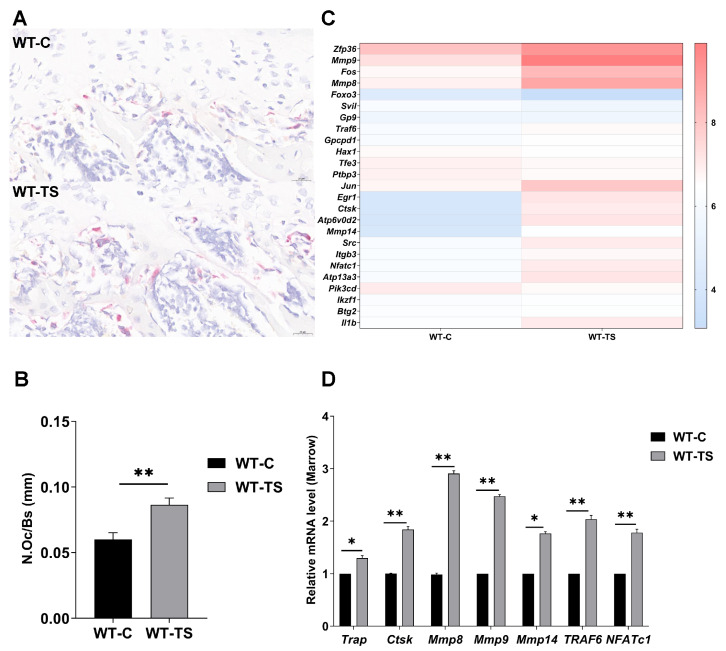
The number of osteoclasts was increased, and the expression of osteoclast-related genes decreased in WT mice after tail suspension. (**A**,**B**) TRAP staining of femur tissue and statistics of osteoclast numbers. (**C**) Cluster heat map of osteoclast expression in RNA-seq, comparison between normal and suspended WT mice. (**D**) RT-qPCR detected the expression of osteoclast-related genes in the bone marrow of WT mice. (All data represent mean ± SD. ** *p* < 0.01, * *p* < 0.05.)

TRAP staining was performed on the femurs of mice from both the regular and supension groups. This was followed by counting the number of osteoclasts present (Figure 2A). Statistical analysis indicated a significant increase in the number of osteoclasts in the femurs of mice from the suspension group compared to the regular group (Figure 2B, *p* < 0.01). This finding suggests that the simulated microgravity environment may promote osteoclastogenesis in mice.

To delve deeper into the mechanism of bone loss under simulated microgravity conditions, bone marrow samples were collected from WT mice in both the regular and suspension groups for RNA-seq analysis. The cluster heatmap generated from this analysis revealed an upregulation of osteoclast-related genes in the simulated microgravity environment (Figure 2C). To corroborate these findings, RT-qPCR was conducted to measure the expression levels of osteoclast-related genes, including *Trap, Ctsk, Mmp8, Mmp9,* and *Mmp14*, along with their regulatory factors *TRAF6* and *NFATc1* (Figure 2D). Compared to the regular group, there was a significant upregulation of these osteoclast-related genes in the suspension group. Consequently, it can be inferred that exposure to a simulated microgravity environment increases both the number and activity of osteoclasts, leading to bone loss in mice.

### 2.3. The Bone Loss in the Simulated Microgravity Environment Were Blocked in Atp6v1h^+/-^ Mice

**Figure 3:**
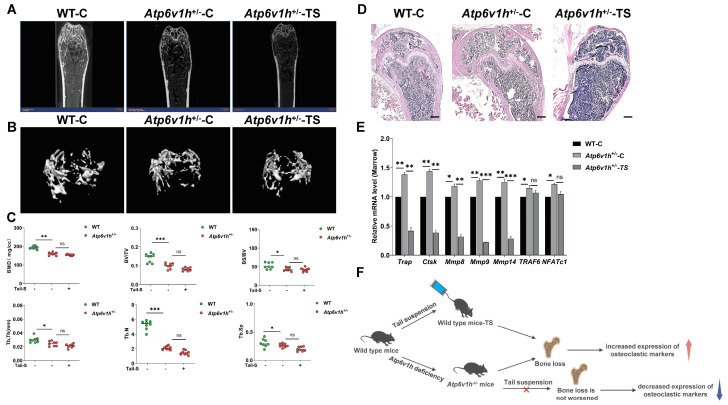
The deficiency of *Atp6v1h* blocks the exacerbation of bone loss under simulated microgravity. (**A**) Two-dimensional image of micro-CT scan of femur. (**B**) Three-dimensional images of micro-CT scan of femur. (**C**) Statistics of micro-CT-related parameters: BMD (bone mineral density), BV/TV (bone volume ratio), BS/BV (bone surface area to bone volume ratio), Tb. Th (trabecular thickness), Tb. N (trabecular number), Tb. Sp (trabecular bone separation) (*** *p* < 0.001, ** *p* < 0.01, * *p* < 0.05, ns: no significant difference). (**D**) H&E staining of mouse femur tissue (scale bar: 200 μM). (**E**) RT-qPCR analysis quantified osteoclast-related gene expression in bone marrow of mice from different groups. (All data represent mean ± SD. *** *p* < 0.001, ** *p* < 0.01, * *p* < 0.05, ns: no significant difference.) (**F**) A schematic diagram, illustrating how *Atp6v1h* deficiency blocks bone loss in mice under simulated microgravity. Each experimental group consisted of 8 mice; all mice were 3 months old.

Femurs from non-suspended WT mice, *Atp6v1h*^+/-^ mice, and suspended *Atp6v1h*^+/-^ mice were analyzed using micro-CT, as shown in Figure 3A,B. Compared to WT mice under normal conditions, *Atp6v1h*^+/-^ mice exhibited significant cortical bone thinning, with a marked decrease in trabecular number, volume, and thickness, and an increase in dispersion, indicating severe bone loss. However, no further exacerbation of bone loss was observed in *Atp6v1h*^+/-^ mice after suspension. As depicted in Figure 3C, compared to WT mice in a normal environment, the *Atp6v1h*^+/-^ mice showed significant reductions in BMD, BV/TV, BS/BV, Tb.Th, Tb.N, and Tb.Sp (*p* < 0.05). Notably, these parameters did not differ significantly before and after suspension in *Atp6v1h*^+/-^ mice. H&E staining of femurs from the three groups, as shown in Figure 3D, further corroborates these findings. In comparison to non-suspended WT mice, *Atp6v1h*^+/-^ mice displayed thinning of cartilage and cortical bone, along with irregular bone morphology; however, no significant changes were observed in *Atp6v1h*^+/-^ mice before and after suspension. This suggests that *Atp6v1h* deficiency may impede bone loss in microgravity conditions. Real-time PCR results, presented in Figure 3E, reveal that, in a normal environment, knockout of *Atp6v1h* in mice led to significant upregulation of osteoclast-related genes, including *Trap, Ctsk, Mmp8, Mmp9, Mmp14, TRAF6,* and *NFATc1* (*p* < 0.05). However, post-suspension, all these genes, except for *TRAF6* and *NFATc1*, showed a significant decrease in expression in *Atp6v1h*^+/-^ mice (*p* < 0.05). Figure 3F provides a schematic representation of the mechanism by which *Atp6v1h* deficiency may block bone loss in mice under microgravity, offering a clearer understanding of the observed results.

### 2.4. Atp6v1h Deficiency Blocks Mice’s Simulated Microgravity-Induced Bone Mineral Density Decrease through Fos-Jun-Src-Integrin Pathway

Our observations demonstrate a marked increase in osteoclast activity in WT mice under simulated microgravity conditions, exacerbating bone loss. Conversely, in *Atp6v1h*^+/-^ mice exposed to the same conditions, osteoclast activity is notably diminished, leading to a mitigation of bone loss. This effect appears to be due to *Atp6v1h* knockout, which blocks osteoclasts’ bone resorption function, thereby slowing the bone resorption process. To elucidate the molecular mechanisms underpinning this pathway, we performed RNA-seq and cluster heatmap analysis of gene expression in bone marrow samples from both WT and *Atp6v1h*^+/-^ mice in regular and suspension groups.

Initial differential gene expression analysis revealed 393 genes differentially expressed between male WT and male *Atp6v1h*^+/-^ groups, 276 genes between the male WT non-suspended and suspended groups, and 196 genes between the male *Atp6v1h*^+/-^ non-suspended and suspended groups. Gene Ontology (GO) biological function enrichment analysis showed significant differences: the male *Atp6v1h*^+/-^ group was enriched in immune system processes compared to the male WT group. The male WT suspended group showed enrichment in cell adhesion, apoptosis, and cell regulation pathways, whereas the male *Atp6v1h*^+/-^ suspended group exhibited enrichment in red blood cell development and iron ion homeostasis. Further analyses, including Kyoto Encyclopedia of Genes and Genomes (KEGG) pathway and gene regulatory network interaction studies, identified several hub genes such as *Jun*, *Fos*, *Itgb5*, *Itgb1*, and *Itgb2*, which require further investigation.

**Figure 4:**
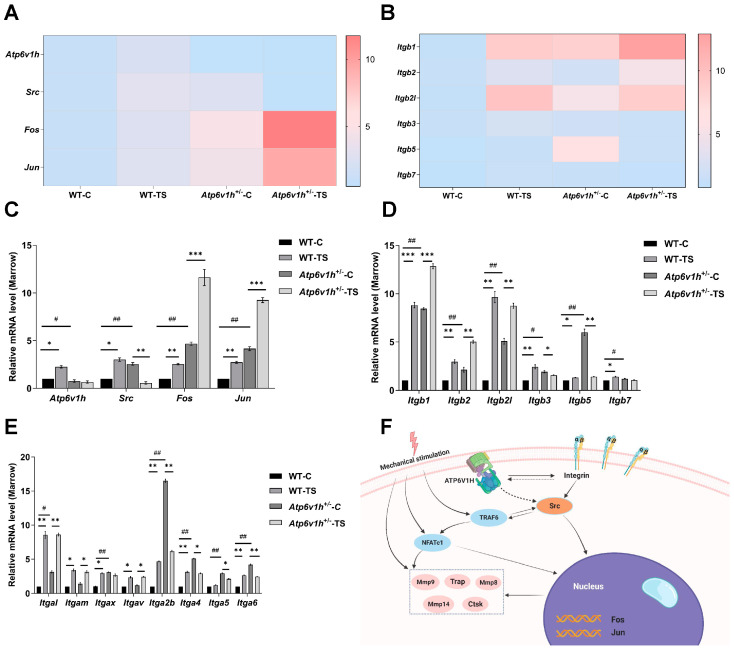
*Atp6v1h* regulates *Fos*, *Jun*, *Src*, and integrin, thereby affecting the expression of osteoclast-related genes. (**A**,**B**) Cluster heat map analysis of integrins in RNA-seq and comparison between the regular and suspension groups in WT mice and *Atp6v1h*^+/-^ mice. (**C**) RT-qPCR detected the expression of *Atp6v1h*-*Fos*-*Jun*-*Src* in mouse bone marrow. (**D**) RT-qPCR analysis of integrin genes expression in the bone marrow of mice across various groups. (**E**) RT-qPCR detected the expression of integrin in mouse bone marrow. (All data represent mean ± SD; “*” represents the significant degree of gene expression changes in mice after suspension, and “^#^” represents the significant degree of gene expression changes in mice after *Atp6v1h* deficiency; *** *p* < 0.001, ** *p* or ^##^ *p* < 0.01, * p or ^#^ *p* < 0.05.) (**F**) Mechanism diagram of *Atp6v1h* regulating the expression of osteoclast-related genes via the *Fos-Jun-Src-Integrin* pathway.

We noted significant changes in the expression of *Fos*, *Src*, *Jun*, and integrin genes like *Itgb1*, *Itgb2*, and *Itgb5* between regular and suspended groups of WT mice (Figure 4A,B). RT-qPCR was conducted for further validation, confirming the increased expression of *Atp6v1h*, *Fos*, *Src*, and *Jun* in WT mice under suspension (Figure 4C,D). Additionally, whereas the expression of *Itga5* remained stable, other integrin subunits’ expression increased, indicating their sensitivity to microgravity. In *Atp6v1h*-deficient mice, expressions of *Fos*, *Src*, and *Jun* were elevated. Except for *Itgam* and *Itgav*, the expression of other integrin subunits also increased, suggesting their regulation by *Atp6v1h* (Figure 4E). This indicates specific relationships of *Itga5* with *Atp6v1h* and *Itgam* and *Itgav* with microgravity. The expressions of other integrin subunits are influenced by both *Atp6v1h* and microgravity. Notably, in the suspension group, the expressions of *Atp6v1h* and *Src* were decreased, whereas *Fos* and *Jun* were increased, alongside mixed changes in various integrin genes’ expressions. This complex interplay of gene expressions, especially under the combined influence of microgravity and *Atp6v1h* knockout, could explain the slowed bone loss in *Atp6v1h*^+/-^ mice under microgravity. Furthermore, *Src* appears to influence *Fos* and *Jun*, which, in turn, regulate osteoclast-related genes (Figure 4F).

### 2.5. Atp6v1h May Directly Regulate the Fos-Jun-Src-Integrin Pathway

**Figure 5:**
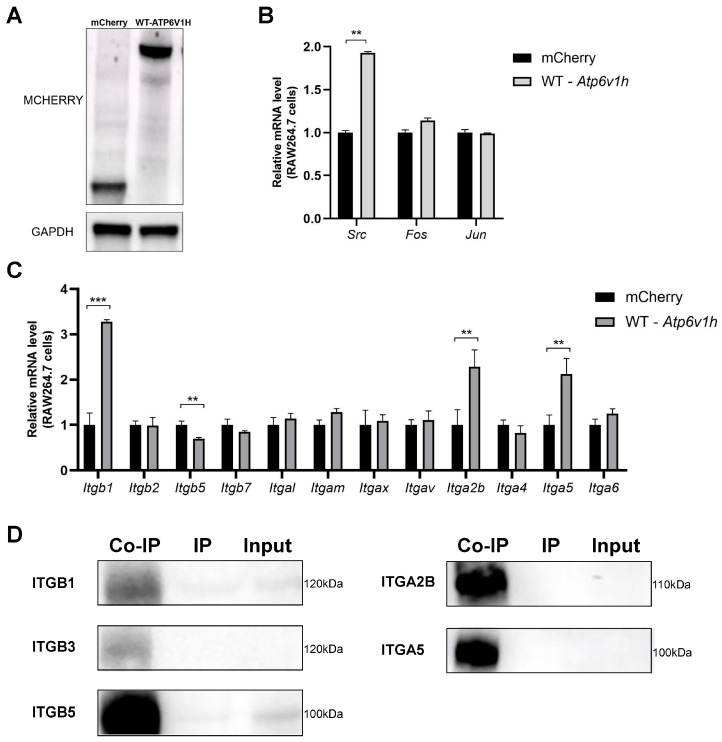
*Atp6v1h* may directly affect the *Fos-Jun-Src-Integrin* pathway. (**A**) RAW264.7 cell transfection *ATP6V1H* was successfully constructed. (**B**,**C**) RT-qPCR was used to detect the expression of genes in the *Fos-Jun-Src-Integrin* pathway in RAW264.7 cells overexpressed with *ATP6V1H*. (**D**) Co-IP detects interactions between ATP6V1H and integrin subunits ITGB1, ITGB5, ITGA2B, and ITGA5. (All data represent mean ± SD. *** *p* < 0.001, ** *p* < 0.01).

To elucidate the specific regulatory relationship between *Atp6v1h* and the *Fos*-*Jun*-*Src*-*Integrin* pathway, we first utilized the RAW264.7 cell line overexpressing *ATP6V1H* (Figure 5A; Supplemental Figure S1). RT-qPCR was employed to assess the expression of molecules within the *Fos*-*Jun*-*Src*-*Integrin* pathway in this cell line. Our analysis revealed a significant increase in *Src* expression following the upregulation of *Atp6v1h*, whereas the expression levels of *Fos* and *Jun* remained relatively unchanged (Figure 5B). This suggests that *Atp6v1h* may specifically regulate the *Fos*-*Jun*-*Src* pathway through *Src* expression, thereby influencing changes in bone mass.

Furthermore, RT-qPCR was used to measure the expression of *integrin* in the RAW264.7 cells overexpressing *Atp6v1h*. We observed that, upon increased *Atp6v1h* expression, there was an upregulation of *Itgb1*, *Itgb3*, *Itga2b*, and *Itga5*, whereas *Itgb5* expression decreased. Other integrin subunit genes did not show significant changes (Figure 5C). These findings indicate that *Atp6v1h* can regulate the expression of these specific integrin subunits. Additionally, when cross-referencing these results with the expression profiles of integrin subunits in the bone marrow of WT and *Atp6v1h*^+/-^ mice, *Itgb1*, *Itgb5*, *Itga2b*, and *Itga5* were found to be influenced by both *Atp6v1h* and microgravity. This suggests that *Atp6v1h* may modulate bone loss in a microgravity environment by regulating these integrin subunits.

To confirm the interaction between ATP6V1H and integrins, Co-IP was performed to detect the binding between ATP6V1H and ITGB1, ITGB3, ITGB5, ITGA2B, and ITGA5 (Figure 5D; Supplemental Figure S2). The results demonstrated a direct association between these five proteins and ATP6V1H. In summary, ATP6V1H is capable of interacting with ITGB1, ITGB3, ITGB5, ITGA2B, and ITGA5. This interaction potentially enables ATP6V1H to regulate downstream osteoclast-related molecules through its influence on SRC, thereby impacting changes in bone mass.

## 3. Discussion

Osteoporosis, a condition characterized by an imbalance between bone resorption and bone formation, is a significant global health concern. Data from the International Osteoporosis Foundation indicates that approximately one in three women and one in five men over the age of 50 experience osteoporotic fractures worldwide. This condition is responsible for an estimated 8.9 million fractures annually, making it one of the most prevalent and serious chronic progressive metabolic bone diseases [22].

The exploration of space, a relentless pursuit since Yuri Gagarin’s historic journey 60 years ago, presents unique challenges to the scientific community, particularly concerning the health of astronauts. One of the pressing issues is understanding and mitigating the harmful effects of space radiation and microgravity on the human body. Research has shown that exposure to microgravity can have detrimental effects on various bodily systems, including the skeletal and muscular systems [23]. Moreover, individuals with different genetic backgrounds may have varying susceptibilities to conditions such as osteoporosis when exposed to these space conditions [24].

Current research on osteoporosis primarily focuses on individual environmental factors or single genes, leaving the interaction between genetics and environmental factors, such as microgravity, and their combined effects on bone loss largely unexplored. Therefore, there is a critical need for comprehensive research involving the development of appropriate animal models. Such studies are essential to investigate the effects of microgravity on individuals with diverse genetic backgrounds, providing insights that could help mitigate bone loss in astronauts and contribute to our understanding of osteoporosis on Earth.

This study represents a significant breakthrough in understanding the complex regulatory mechanisms of environmental and genetic factors on bone health. It specifically focuses on the bone-related gene *ATP6V1H*, previously identified by our research group, and its interaction with microgravity environments. *ATP6V1H*, known to be highly expressed in lysosomes, influences bone integrity by affecting osteoclast production and function [25,26]. Our prior research, including immunohistochemical staining and TRAP staining, revealed that *ATP6V1H* localizes in osteoclasts. Notably, osteoclasts in heterozygous knockout mice exhibited relatively weak positive staining of *Atp6v1h*. Jiang et al.’s research further substantiated that ATP6V1H can promote osteogenic differentiation of MC3T1-E3 cells through the Akt/GSK3β signaling pathway [27].

Building upon our group’s preliminary findings on the molecular mechanisms by which ATP6V1H regulates bone homeostasis, this study aims to further investigate the potential interactive regulatory effects of external environmental factors and varying genetic backgrounds on bone density. This inquiry seeks to discern whether external influences and genetic predispositions synergistically or antagonistically impact bone density, thereby contributing to a more comprehensive understanding of bone physiology under diverse conditions. Further transcriptome analysis of different experimental groups suggested that the milder bone loss in *Atp6v1h*^+/-^ mice, exposed to the same tail suspension as WT mice, might be intricately linked to integrins. Integrins, transmembrane receptors, facilitate cell–extracellular matrix interactions and are composed of alpha (α) and beta (β) subunits. To date, 18 α and 8 β subunits have been identified, forming over 20 different integrins [28]. Key integrins on osteoclast membranes include αvβ3, α2β1, and αvβ1. β1 integrin mediates osteoclast binding to fibronectin, whereas αvβ3 is crucial for osteoclast adhesion to bone sialoprotein and osteopontin, with its active form promoting osteoclast migration and bone resorption [29,30].

Sanjay et al. have shown that the PyK2/Src/Cbl molecular complex regulates osteoclast movement and adhesion. Activation of αvβ3 integrin triggers Ca^2+^-dependent phosphorylation of Pyk2 at Y402, facilitating its association with Src SH2 and subsequent Src activation. This cascade leads to the recruitment and phosphorylation of c-Cbl, dependent on Src SH3. Interestingly, the PTB domain of Cbl binds to the phosphorylated Tyr-416 within Src’s activation loop, inhibiting Src kinase activity and, consequently, integrin-mediated adhesion [31].

In the current study, integrin subunits displayed heightened expression levels in both transcriptome sequencing and RT-qPCR analyses. Furthermore, these expression levels underwent significant changes in response to microgravity conditions or *Atp6v1h* knockout. It is well-documented that integrins can respond to mechanical cues in the extracellular environment, effectively transmitting mechanical signals to cells [32].

To further decipher the regulatory relationship between ATP6V1H and integrin, we employed the Co-IP method. The results indicated that ATP6V1H directly interacts with the integrin subunits ITGB1, ITGB3, ITGB5, ITGA2B, and ITGA5. These findings suggest that ATP6V1H may modulate osteoclast function and thereby influence bone resorption through its interaction with integrin. However, the specific mechanisms underlying these interactions warrant additional investigation.

As a downstream effector of integrin, Src, a tyrosine kinase, plays a pivotal role in regulating bone metabolic balance. It influences the formation of bone lacunae seal zones and folding boundaries [33]. Integrins, when stimulated mechanically, can transmit signals to downstream molecules like Src [34]. In our study, we observed that integrin regulates changes in Src expression under microgravity conditions. Src, in turn, modulates the expression of downstream bone-related molecules and facilitates bone resorption. Notably, both integrin and Src expressions increased under individual conditions of microgravity environment or *Atp6v1h* knockout. However, when combined, these factors led to a decrease in integrin and Src expressions. This underscores Src’s vital role in mediating the combined effects of microgravity and *Atp6v1h* knockout.

In summary, this research integrates environmental and genetic factors to explore how the microgravity environment and *Atp6v1h* knockout regulate osteoclast activity via the *Fos-Jun-Src-Integrin* pathway, affecting bone loss in microgravity conditions. These insights lay the groundwork for potential ATP6V1H-based interventions in treating bone loss under microgravity. It is important to note, however, that while this study has established a preliminary connection between ATP6V1H and integrin, further research is needed to fully understand the specific mechanisms, including whether direct complex formation or intermediary molecules are involved.

## 4. Materials and Methods

### 4.1. The Animals Utilized for This Study

The WT C57BL/6J mice were purchased from Cyagen, China. The *Atp6v1h* knockout mice utilized in this study were previously generated by our research group through the application of CRISPR/Cas9 technology. All mice were housed in a standard animal facility, with conditions meticulously maintained at 20 ± 2 °C and 50 ± 10% relative humidity, under a 12 h light–dark cycle. Both WT and tail-suspended mice were pair-fed and given free access to food and water. Subsequently, specific breeding protocols were established for the propagation of WT C57BL/6J and *Atp6v1h* knockout mice.

The *Atp6v1h* gene in mice, targeted for knockout using CRISPR/Cas9, involved a frame shift mutation in exon 2. This was achieved using a specific gRNA, constructed with a Church Lab vector and the mMESSAGE mMACHINE T7 Kit. The modified genes were injected into C57BL/6 mouse eggs, with genotypic changes confirmed through PCR genotyping and sequencing. For detailed procedures on the construction of *Atp6v1h*^+/-^ mice, please refer to reference [18]. All animal experiments conducted in this study received approval from the Laboratory Animal Care & Welfare Committee, School of Stomatology, Fourth Military Medical University (Approval Date: 1 March 2015; Project ID: 81470728). These experiments were carried out in strict compliance with the principles outlined in the 1964 Helsinki Declaration, along with its subsequent amendments, ensuring ethical and responsible research practices.

### 4.2. Construction and Culture of Overexpressing ATP6V1H Cells

We engaged Cyagen, China, to construct both the overexpressing WT-*ATP6V1H* lentivirus vector and the mCherry lentivirus vector for infecting RAW264.7 cells. Cells infected with the mCherry lentivirus served as the negative control. Initially, normal RAW264.7 cells were cultured in 6-well plates at a density of 1 × 10^6^ cells. Within 12–24 h of incubation, we introduced lentivirus at an MOI of 25, along with a medium containing 5 μg/mL polybrene and 0.5 mL DMEM. After 6–8 h, this was supplemented with a complete medium (DMEM medium + 10% fetal bovine serum + 1% Penicillin-Streptomycin). Post-48 h, we selected for puromycin-resistant cells using 2 μg/mL puromycin. The medium was refreshed bi-daily until stable puromycin-resistant cell lines were established.

### 4.3. Tail Suspension Model

Our study utilized a modified mouse tail suspension model, based on National Aeronautics and Space Administration (NASA) and the International Society of Zoological Sciences (ISZS) recommendations, with reduced cage size and adjustable screws on both cage and feeder sides, plus a pulley system on the tail suspension bracket for sufficient movement range [35]. Three-month-old WT and *Atp6v1h*^+/-^ mice were randomly assigned to either the non-suspension or suspension groups, each comprising eight mice. The suspended mice’s tails were attached to the top of the bracket, positioned head-down with hind legs at a ~30-degree angle from the base, for 28 days. The suspension height was adjustable to ensure hind legs did not touch the ground and allowed normal feeding.

### 4.4. Micro-CT Scanning and Analysis

On the 28th day of the experiment, all 8 mice in each group were euthanized using excessive anesthesia, and their femurs were dissected. These specimens were initially fixed in 4% paraformaldehyde, followed by preservation in 75% ethanol, before being scanned with micro-CT. Scanning was performed using an Explore Locus SP micro-CT system at 80kV and 500mA. Reconstruction and analysis of the scans were conducted using Health Care MicroView ABA 2.1.2 software. The regions of interest included: a trabecular bone test area located 2mm below the growth plate and a cortical bone test area 4mm below the growth plate. Parameters such as femur bone mineral density and other bone characteristics were computed and compared. The trabecular morphology was assessed through variables like bone volume/total volume (BV/TV), trabecular thickness (Tb. Th), trabecular number (Tb. N), and trabecular separation (Tb. Sp). Cortical bone metrics included total cross-sectional area inside the periosteal envelope (Tt. Ar), cortical bone area (Ct. Ar), cortical area fraction (Ct. Ar/Tt.Ar), and average cortical thickness (Ct. Th). By analyzing these indices, which reflect the internal morphological changes in the femurs, we aimed to discern the combined impact of *Atp6v1h* knockout and simulated microgravity on the mice.

### 4.5. Bone Tissue Staining

The femurs of 8 mice in each experimental group were subjected to decalcification in 10% EDTA (pH 7.2–7.4) at 37 °C for 20–30 days, followed by a gradient ethanol dehydration process and embedding in paraffin. The sections were cut to a thickness of 4–5 μm. These sections were stained using H&E, sourced from the Nanjing Jiancheng Institute of Bioengineering, Nanjing, China. Microscopic examination of the distal femoral regions was conducted using a Leica Microsystems DMI6000B Microscope (Wetzlar, Germany), allowing for detailed analysis of the internal microstructure of femoral trabecular bones.

Osteoclast identification in the sections was achieved through staining with a commercial kit (CS0740, Sigma, St. Louis, MO, USA). Tartarate-resistant acid phosphatase (TRAP)-positive osteoclasts located adjacent to the bone surface were quantified in accordance with standardized counting procedures.

### 4.6. RNA-Sequencing Analysis

In each experimental group, femurs and tibias were collected from mice. Each group comprised three samples, totaling 24 samples across all groups. The bone marrow was extracted by flushing with phosphate-buffered saline (PBS) after the removal of proximal and distal epiphyses. Following this, red blood cell lysis buffer was added, and the suspension was incubated for 5 min. Bone marrow mRNA was isolated utilizing the Total RNA Kit (Cwbio, Beijing, China). The quality and quantity of the RNA were assessed using a NanoDrop spectrophotometer and an Agilent 2100 bioanalyzer (Thermo Fisher Scientific, Waltham, MA, USA). Subsequently, transcriptome sequencing was performed, generating 100 base pair paired-end reads on the DNBseq platform, targeting 50 million reads per sample. The sequencing reads were aligned to the human genome using Rsubread (Bioconductor) with reference to GENCODE GRCh38.p13. Aligned reads were quantified with featureCounts (Bioconductor), excluding chimeras, multi-mapping genes, multi-overlap, and single-end genes. Differential expression analysis was conducted using edgeR (Bioconductor), adhering to the following criteria: counts > 10, *p* < 0.05, |log2(Fold Change) | > 1, and a false discovery rate (FDR) < 0.05. The gene expression heatmap was created utilizing the “heatmap” function available in the NMF package of R (version 4.2.2).

### 4.7. Gene Expression by RT–qPCR

As described in Section 2.5, total RNA was isolated from the bone marrow of male mice. This RNA was subsequently reverse transcribed into cDNA utilizing the PrimeScript™ RT Reagent Kit. To quantify the mRNA expression of *Atp6v1h*, *Itgav*, *Itgb3*, *Src*, and various osteoclast-related genes, real-time PCR was performed. This analysis employed SYBR^®^ Premix Ex Taq™ (TaKaRa) on an ABI 7500 real-time PCR system (Applied Biosystems). Primer specifics are detailed in Table 1. Glyceraldehyde 3-phosphate dehydrogenase (*Gapdh*) served as the internal control, and its threshold cycle (Ct) values were used to normalize the data. Relative mRNA levels were determined through three independent experiments. The expression levels of the PCR products were calculated using the comparative CT (ΔΔCT) method.

### 4.8. Co-Immunoprecipitation

The study involved various RAW264.7 cell derivatives: native RAW264.7 cells, cells transfected with an empty vector, cells overexpressing *ATP6V1H*, and cells with mutated *ATP6V1H*. These cell lines were cultured, enumerated, and subsequently harvested. After an incubation period of 8 h, cells were lysed using RIPA lysis buffer (Proandy, Xi’an, China), followed by centrifugation at 12,000× *g* for 15 min to collect the cell protein supernatant. Co-immunoprecipitation (Co-IP) experiments were conducted using a commercial kit (Beaver, Suzhou, China), strictly following the manufacturer’s instructions. Initially, magnetic beads were conjugated with ATP6V1H antibodies. Subsequently, these antibody-bound beads were incubated with the cell protein supernatant to facilitate the antigen-antibody binding. The antigen-bound complex was then eluted and stored at −80 °C for subsequent analyses.

### 4.9. Western Blot

The protein concentration in the Co-IP samples was quantified using the BCA Protein Assay Kit (Cwbio, Beijing, China). For electrophoretic analysis, 30 µg of protein from each sample was mixed with 5× SDS-PAGE loading buffer and denatured at 95 °C for 5 min in boiling water. The proteins were then separated by SDS-PAGE and subsequently transferred onto polyvinylidene fluoride (PVDF) membranes (Millipore, Billerica, MA, USA). These membranes were blocked using 5% non-fat dry milk in TBST for one hour, followed by overnight incubation at 4 °C with primary antibodies. The primary antibodies used were rabbit anti-Atp6v1h (1:500), rabbit anti-integrin Beta 1 (1:400), rabbit anti-integrin Beta 3 (1:2000), rabbit anti-integrin Beta 5 (1:3000), rabbit anti-integrin Alpha 2B (1:3000), and rabbit anti-Alpha-5 (1:3000), all sourced from Proteintech Group, Wuhan, China.

Following this, the membranes were washed thrice with TBST and incubated with a goat anti-rabbit secondary antibody (1:10,000, Proteintech Group, Wuhan, China) for 50 min. The protein bands were visualized and captured using a chemiluminescence detection system (VILBER). Quantitative analysis of the protein bands was performed using AccuRef Scientific software (Xi’an, China).

### 4.10. Statistical Analysis

Statistical analyses of the data were performed using GraphPad Prism version 8.0 (GraphPad Software, La Jolla, CA, USA). To examine the relationships between weight data groups, correlation analysis was employed. Within-group differences were assessed using analysis of variance (ANOVA). The Student’s t-test was applied for the comparison of bone parameters and other relevant factors between groups. Furthermore, two-way ANOVA was utilized to investigate differences in bone mass between male and female mice across various genotypes after suspension.

Statistical significance was determined based on *p*-values, with a *p*-value less than 0.05 deemed significant. The levels of significance were denoted as follows: * *p* < 0.05, ** *p* < 0.01, and *** *p* < 0.001, indicating increasingly significant differences compared to the control groups.

## 5. Conclusions

Our research indicates that *Atp6v1h* plays a crucial role in regulating bone loss in microgravity conditions through the *Fos-Jun-Src-Integrin* pathway. This pathway influences osteoclast activity and bone resorption, which are key factors in the development and progression of osteoporosis. Consequently, manipulating the expression of *Atp6v1h* presents a viable strategy to control bone loss in environments characterized by microgravity.

Furthermore, this study has elucidated the molecular mechanisms by which *Atp6v1h* contributes to osteoporosis. By demonstrating that interventions targeting *Atp6v1h* can effectively mitigate bone loss, our findings provide important insights into novel therapeutic approaches for osteoporosis. These discoveries not only advance our understanding of bone metabolism in altered gravity conditions but also offer promising avenues for the development of treatments to combat osteoporosis.

## Figures and Tables

**Table 1:** Sequences of primers used for RT–qPCR.

Gene Name	Forward Primer (5′-3′)	Reverse Primer (5′-3′)	PCR Product Size (bp)
*Gapdh*	CATGTTCCAGTATGACTCCACTC	GGCCTCACCCCATTTGATGT	136
*Atp6v1h*	GGATGCTGCTGTCCCAACTAA	TCTCTTGCTTGTCCTCGGAAC	164
*Src*	TCTGAACCAAGGCAGCATCT	TGGCCTAAAGACCCTGTTGC	108
*Fos*	AGAGCGGGAATGGTGAAGAC	AGTTGATCTGTCTCCGCTTGG	194
*Itgb1*	CAGACTTCCGCATTGGCTTT	TGTGCCCACTGCTGACTTAG	546
*Itgb2*	CCCACTGGCCTAGAACTTCA	CCTGTCCTGGTCGCAAGTAA	200
*Itgb2l*	CCAATACCACCATCCTACTGGG	TCTTGGAAATGAGGCTGCTGTG	163
*Itgb3*	CTCCCCTCCGCAGGAAAA	ATCTTCGAATCATCTGGCCGTA	448
*Itgb5*	GCCCGTTATGAAATGGCCTC	CTACCAGGTCCCTTAGGGCT	247
*Itgb7*	CCATGTGAGCAGTACAGGGAT	GGGTGTGATCCACTCCCTTC	241
*Itgal*	TCTCACTACTTTCCGGATCCCT	AGTAGGCATCTTCCCCTGAGT	222
*Itgam*	AGAGGCTCTCAGAGAATGTCCT	TCTCACTACTTTCCGGATCCCT	295
*Itgax*	GCATGTCATAACAGAACTGTTCACC	TGGTGGGAAACCATCTTTGTGT	190
*Itgav*	CTTCATTACGACATTGACGGGC	GGATTTCACGTACAGGATTGCG	258
*Itga2b*	ACCTCAGGGATGAGACACGA	GCTTGTCTCCGCGAAAACTC	279
*Itga4*	GGTCCCAGGCTACATCGTTTT	ATGCCCAAGGTGGTATGTGG	232
*Itga5*	GGTTGCCTGAGTTCCATCCA	CCAGAATCCGGGAGCCTTTG	194
*Itga6*	CGGTCCAGACAGATGTCCAC	CTTGGATCCGAAGGGCTGTT	217
*Jun*	GGGAGCATTTGGAGAGTCCC	TTTGCAAAAGTTCGCTCCCG	182
*NFATc1*	CGTGTCGGCAAAGGAGAGG	CACATAACTGTAGTGTTCTTCCTCG	251
*TRAF6*	GATCCAGGGCTACGATGTGG	CTTGTGCCCTGCATCCCTTA	157
*Trap*	AAAATGCCTCGAGACCTGGG	CACACCGTTCTCGTCCTGAA	243
*Ctsk*	GTTCCTGTTGGGCTTTCAGC	CCGTTCTGCTGCACGTATTG	169
*Mmp 8*	CCTACCCAACGGTCTTCAGG	ACTCCTGGGAACATGCTTGG	288
*Mmp 9*	TGGTCTTCCCCAAAGACCTG	CACAGCGTGGTGTTCGAATG	216
*Mmp 14*	GGCGGGTGAGGAATAACCA	GTCTTCCCATTGGGCATCCA	256

## Data Availability

The data that support the findings of this study are available from the corresponding author upon reasonable request.

## Supplementary Materials

The following supporting information can be downloaded at: https://www.mdpi.com/article/10.3390/ijms25010637/s1.
