# Supplementary figures and images for: Atp6v1h Deficiency Blocks Bone Loss in Simulated Microgravity Mice through the Fos-Jun-Src-Integrin Pathway
Source: Int J Mol Sci. 2024 Jan 4;25(1):637. doi: 10.3390/ijms25010637 (PMC10779874; doi:10.3390/ijms25010637)

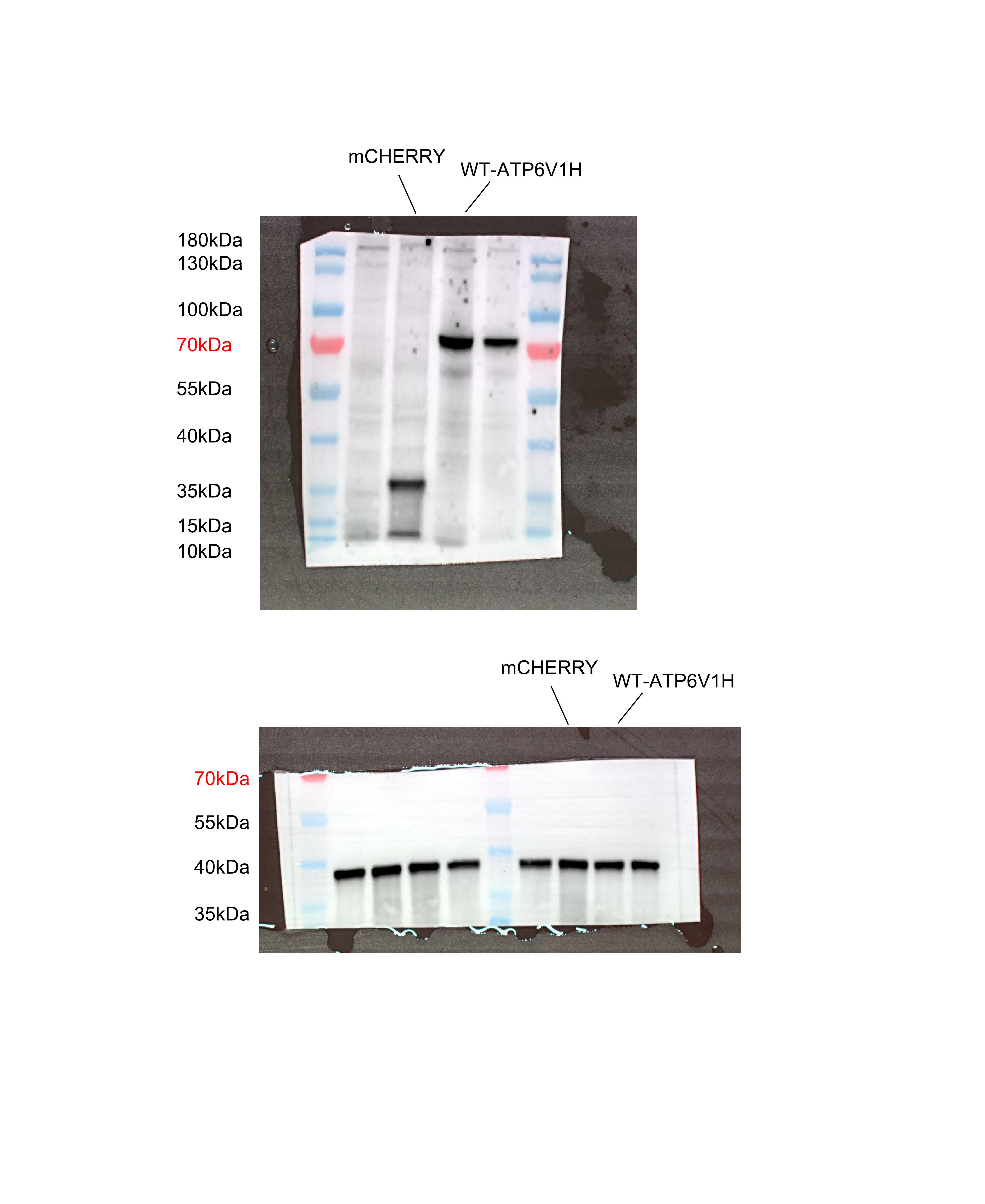

Supplement: Supplementary file 1 [file ijms-25-00637-s001.zip › Supplementary material/Supplemental Fig. S1.tif]

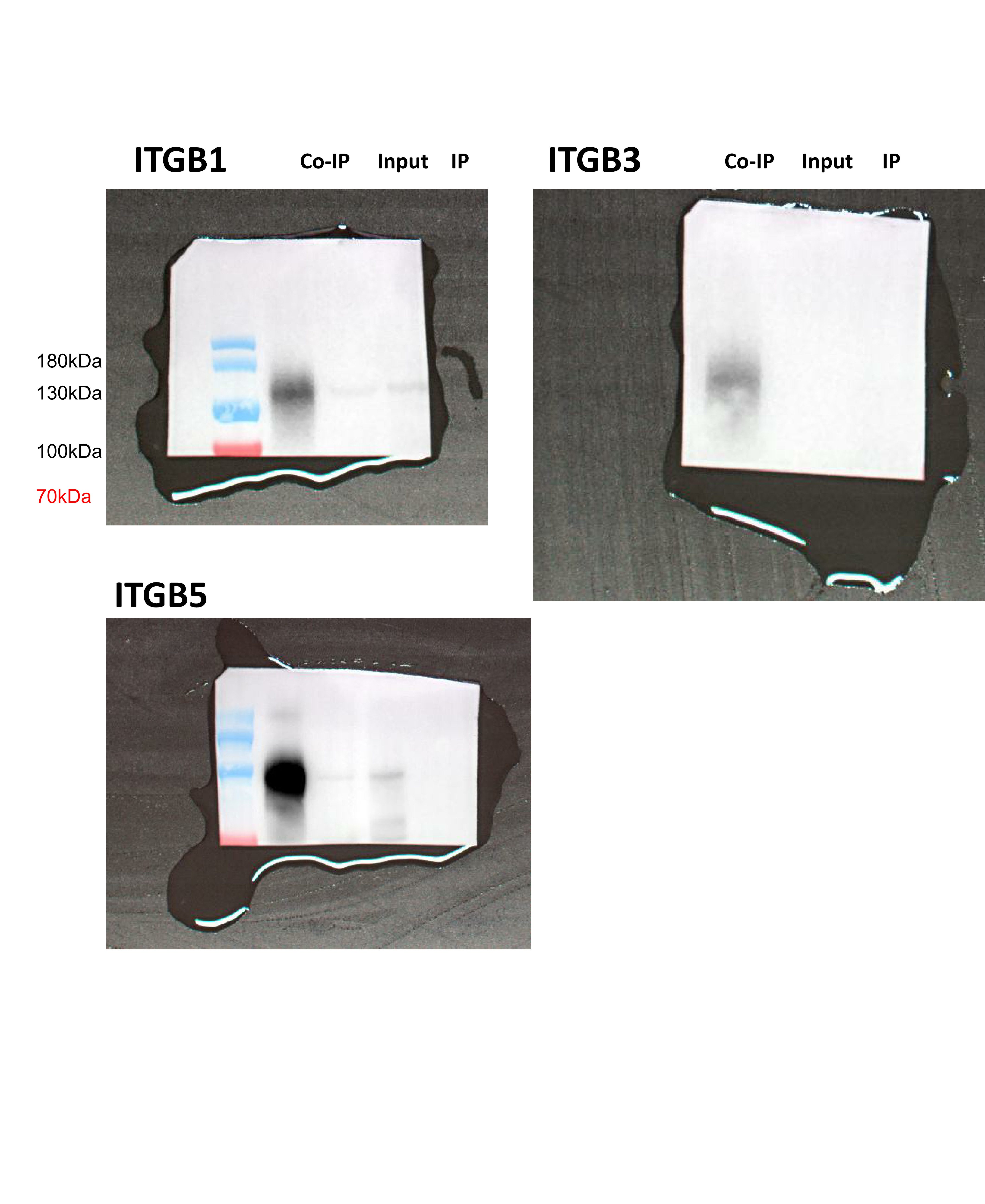

Supplement: Supplementary file 1 [file ijms-25-00637-s001.zip › Supplementary material/Supplemental Fig. S2 (1).tif]

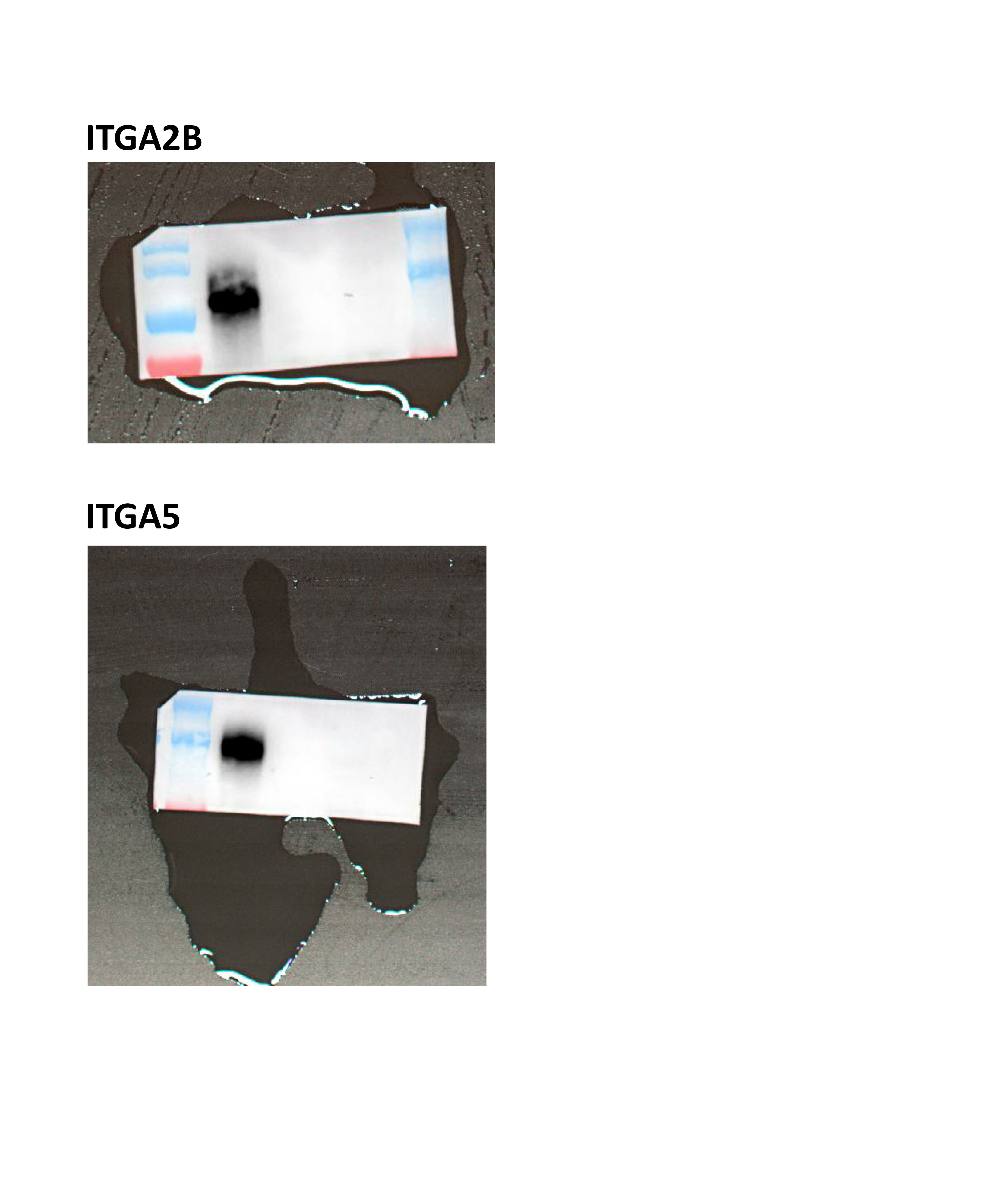

Supplement: Supplementary file 1 [file ijms-25-00637-s001.zip › Supplementary material/Supplemental Fig. S2(2).tif]
